# A Rare Case of Spontaneous Cardiac Tamponade Induced by Concomitant Use of Rivaroxaban and Amiodarone

**DOI:** 10.1155/2018/1650716

**Published:** 2018-08-12

**Authors:** Oreoluwa Oladiran, Jared Segal, Ifeanyi Nwosu, Salik Nazir

**Affiliations:** ^1^Reading Hospital, Tower Health System, Reading, PA, USA; ^2^Benenden Hospitals Trust, Kent, UK

## Abstract

Rivaroxaban is a direct oral anticoagulant (DOAC) approved as an important alternative to warfarin in patients with nonvalvular atrial fibrillation. We report the case of an 87-year-old man with past medical history of nonvalvular atrial fibrillation on rivaroxaban and recently started amiodarone for pulseless ventricular tachycardia who presented to our hospital with intermittent chest pain and was diagnosed with spontaneous hemopericardium causing cardiac tamponade. The culprit drugs were discontinued, and the patient was treated with emergent pericardiocentesis. Both rivaroxaban and amiodarone are substrates for the CYP3A4 hepatic pathway, and concomitant use can result in increased plasma rivaroxaban levels causing an increased propensity to bleeding. While most physicians are cognizant of the need for renal dosing of rivaroxaban, this article aims to increase awareness of its interactions with drugs that are also metabolized through the same hepatic CYP450 pathway.

## 1. Background

Rivaroxaban is a direct oral anticoagulant approved by the United States Food and Drug Administration (FDA) for stroke prevention in nonvalvular atrial fibrillation [1]. Amiodarone is an antiarrhythmic agent indicated for the prevention of recurrent life-threatening ventricular arrhythmias [2]. All anticoagulants including rivaroxaban are associated with increased risk of bleeding. The risk of bleeding with rivaroxaban can be accentuated by the concomitant use of medications such as amiodarone [3] which compete for metabolism via the CYP3A4 hepatic pathway [4]. The incidence of spontaneous hemopericardium due to rivaroxaban is unknown, likely due to its rarity as only few cases have been documented so far [5–7]. However, with increasing use of this medication, rare side effects like this will be encountered more often. Clinicians must be aware of this rare catastrophic possibility that could result from the lethal combination of rivaroxaban and amiodarone. We suggest that rivaroxaban package insert should include more information on medications considered to be inhibitors of CYP3A4 and P-glycoprotein system.

## 2. Case Presentation

An 87-year-old male with medical history of paroxysmal atrial fibrillation on 20 mg of rivaroxaban daily, recent pulseless ventricular tachycardia with implantable cardiac defibrillator in situ, and nonischemic cardiomyopathy (ejection fraction 35%) presented to the emergency room (ER) with intermittent chest pain and light headedness of 2 days duration. Chest pain was exertional, left sided, and pleuritic. He also reported shortness of breath but denied cough, fever, or any other infectious. Of note, he was commenced on 200 mg amiodarone daily four months prior to presentation following an episode of syncope due to pulseless ventricular tachycardia.

Initial vital signs showed blood pressure of 89/60 mmHg with pulse rate of 59/min, temperature of 98.2°F, and respiratory rate of 14 breaths/min with normal oxygen saturation of 100% on ambient air. On physical examination, he was in no acute distress and was alert and oriented to time, place, and person. Jugular venous distention was noted on neck examination. Heart sounds were muffled, but lung fields were clear to auscultation. Peripheral pulses were also weak but palpable.

## 3. Investigations

**Figure 1:**
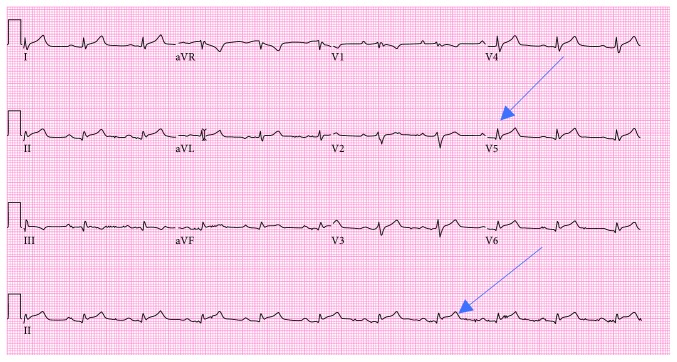
Electrocardiogram showing diffuse ST segment elevation with low voltage QRS complexes.

His EKG on arrival to the emergency room showed new widespread ST segment elevation (Figure 1). His troponin was trended and remained <0.03 ng/mL (normal <0.05 ng/mL). Complete blood count showed hemoglobin of 11.2 g/dL (normal 14–17.5 g/dL), white blood cell count of 11.7/microliter (normal 4.8–10.8/microliter), and normal platelet count of 211/microliter (normal 130–400/microliter). Basic metabolic panel revealed creatinine of 3.4 mg/dL from baseline of <0.8 mg/dL (normal 0.6–1.3 mg/dL) with BUN of 58 mg/dL (normal 7–25 mg/dL). His liver function test showed AST of 90 U/L (normal 13–39 U/L), ALT of 126 U/L (normal 7–52 U/L), and total bilirubin of 1.2 mg/dL (0.3–1.0 mg/dL). INR was also elevated at 2.2 (normal 0.9–1.1) with prothrombin time of 24.7 seconds (normal 12.3–14.6 seconds). Initial lactate was also elevated at 3.2 meq/L (normal 0.6–1.4 meq/L).

**Figure 2:**
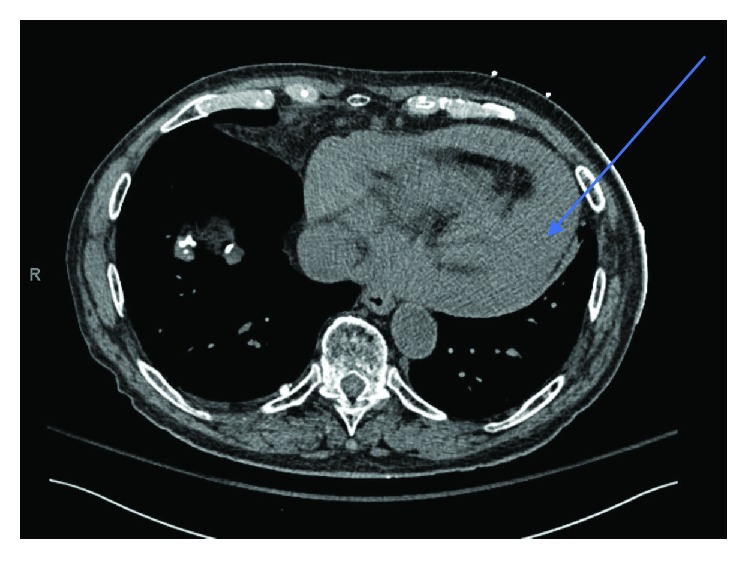
Chest CT scan (transverse view) showing fluid in the pericardial space.

Chest X-ray revealed enlargement of the cardiomedistinal silhouette. CT chest revealed new moderate-sized pericardial effusion (Figure 2).

**Figure 3:**
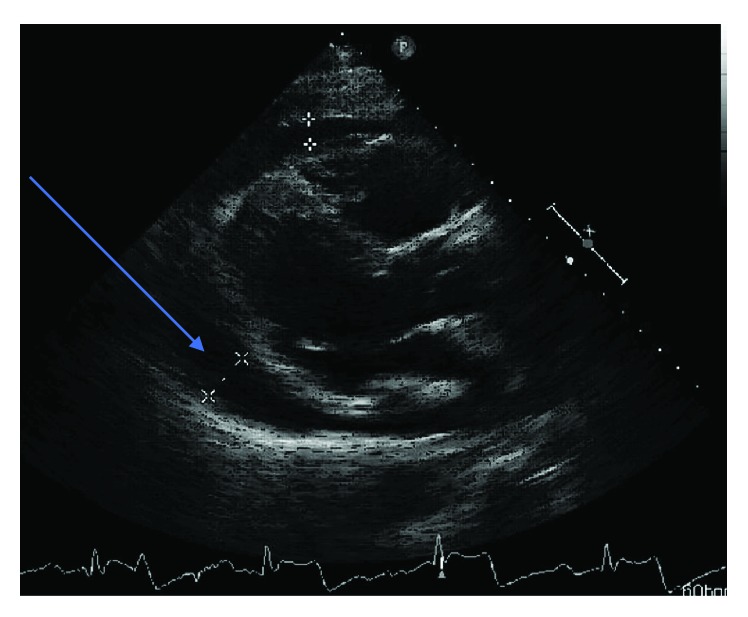
Parasternal long axis view of transthoracic echocardiogram showing fluid within the pericardial space.

Transthoracic echocardiogram (TTE) revealed large circumferential pericardial effusion and evidence for tamponade physiology with multichamber collapse and significant respirophasic variation to the mitral inflow pattern (Figure 3). Please see Supplementary Material (available here).

## 4. Differential Diagnosis

Given the patient's history of intermittent chest pain and presence of ST segment elevation on EKG, our initial suspicion was an ST segment elevation myocardial infarction. However, at the time of evaluation by the cardiologist, the patient had no chest pain and serial troponin levels remained normal. Notably, patient had coronary angiography done 9 months prior to index presentation revealed minimal plaquing of the coronary arteries. We now know that the hypotension, muffled heart sounds, and jugular venous distention were part of the Beck's triad of cardiac tamponade. The lactic acidosis, acute kidney injury, and elevated liver enzymes were likely the result of tissue hypoperfusion from hypotension while the leucocytosis was likely reactive.

## 5. Treatment

Based on the initial finding of ST segment elevation on EKG, he received a loading dose of 324 mg of ASA by EMS. Amiodarone was discontinued due to deranged liver and renal functions. We also held his rivaroxaban due to abnormal clotting profile and finding of hemopericardium on imaging studies. He had urgent pericardiocentesis in the catheterization laboratory with initial drainage of 825 mLs of sanguinous fluid, and postprocedural TTE showed evidence of resolution of tamponade. The pericardiocentesis catheter was left in place to drain any residual collections, and patient was transferred to the intensive care unit. He had daily TTE at the bedside, and his pericardial drainage volume progressively reduced from 160 cc on day 1 to 100 cc on day 2 to 80 cc on day 3 postpericardiocentesis when the drain was removed. Pericardial fluid analysis was negative for organisms or malignancy.

## 6. Outcome and Follow-Up

Our patient experienced significant improvement of his symptoms following pericardiocentesis. His blood pressure normalized, and IV dopamine was discontinued immediately after pericardiocentesis. His renal function also improved significantly evidenced by a reduction in creatinine to normal at the time of discharge. He went home 5 days after admission and was instructed to stop his amiodarone and rivaroxaban in the mean time until his office appointment with his cardiologist.

## 7. Discussion

Based on the Naranjo causality assessment scale [8], there is probable relationship between our patient's spontaneous hemopericardium and rivaroxaban use.

Serious bleeding events have been reported with rivaroxaban, mainly intracranial and gastrointestinal [9], but only a few case reports of nontraumatic hemopericardium exist.

Altered hepatic metabolism resulting from coadministration with amiodarone and transient renal impairment synergistically resulted in supratherapeutic serum levels of rivaroxaban in our patient resulting in nontraumatic hemopericardium and eventual cardiac tamponade.

Elimination of rivaroxaban occurs through a dual pathway—hepatic metabolism occurs via the CYP450 pathway (mainly by CYP3A4 and CYP2J2) while renal excretion via P-glycoprotein-mediated secretion [4]. Amiodarone and its main metabolite N-desethylamiodarone (NDEA) inhibit CYP3A4 and P-glycoprotein resulting in supratherapeutic concentrations of rivaroxaban when used simultaneously.

When used for stroke prophylaxis in patients with nonvalvular atrial fibrillation, clinical trials evaluating the safety and efficacy of rivaroxaban using the cockroft-gault formula recommend that for creatinine clearance greater than 50 mL/minute as was the case in our patient prior to this admission, a standard dose of 20 mg once daily is adequate. However, for CrCl between 15 and 50 mL/minute, a dose adjustment to 15 mg once daily may be considered. Most clinicians recommend discontinuing rivaroxaban in acute kidney injury or CrCl less than 15 mL/minute [9]. No guidelines exist for dose adjustment when used with other medications undergoing CYP450 metabolism.

Andexanet alfa, a recombinant modified human factor Xa protein, is an antidote recently approved by the United States Food and Drug Administration (FDA) [10] for patients treated with rivaroxaban and apixaban when reversal of anticoagulation is needed for life-threatening bleeding [11].

However, there are neither routinely available laboratory tests to measure the anticoagulant effect of rivaroxaban nor consensus guidelines on the use of prothrombin complex concentrates, recombinant factor VIIa, and the newly approved reversal agents in patients on direct oral anticoagulants.

This article aims to alert clinicians to this rare but increasingly reported side effect of rivaroxaban. A high index of suspicion is needed for recognition and diagnosis of spontaneous hemopericardium. Caution should be observed especially in elderly patients with declining renal function and increased likelihood of polypharmacy. With increasing use of rivaroxaban and other novel anticoagulants and the recent approval of a new reversal agent, more research is needed to develop monitoring laboratory parameters to determine and monitor their therapeutic range.

## Supplementary Materials

Supplementary Materials Parasternal long axis view of transthoracic echocardiogram showing large circumferential pericardial effusion with evidence for tamponade physiology.
